# Telling Your Research Story

**Published:** 2024

**Authors:** Houmam Araj

**Affiliations:** 1 Department of Health and Human Services, National Eye Institute/NIH, Bethesda, MD 20817, USA

## Perspective

It is impossible for a person to begin to learn what he thinks he already knows. That’s what Epictetus, the Greek Stoic philosopher, taught us two millennia ago. Yet somehow that lesson never made it to some of us – myself included. Rather, we have to learn it on our own and pay the appropriate price.

So, consider this. How well do you think you know peer review? As a grant applicant? As a paper author? Even as a reviewer for a funding agency or a journal? What do you think are the elements of a successful submission?

My NIH colleagues, Leroy Worth, Jr, from the National Institute of Environmental Health Sciences (NIEHS), Dave Yeung, from the National Institute of Allergy and Infectious Diseases (NIAID), and myself from the National Eye Institute (NEI) tackled this question by summarizing our over 60 years of experience with the NIH program and review processes. Our contribution has been recently published in Proceedings of the National Academy of Sciences (PNAS) [1].

We focused on NIH grant application submissions – although not surprisingly, a number of the elements we describe also apply to successful journal submissions since the two goals substantially overlap. We also strived to keep our views candid. Essentially, this is the advice we would follow if we were submitting our own grant application.

Equally important, we aimed to ensure that our roadmap was both comprehensive and accessible, while also making it engaging and enjoyable to read.

Since imitation is the highest form of flattery – we used Euclid’s masterpiece *“The Elements”* as our model to organize and lay out what we learned. Euclid had 13 Books. We have 13 Books. Euclid had 5 postulates. We have 5 postulates. Euclid had propositions. We have propositions. Euclid had timelessness. We have … ahh, never mind.

We summarized our advice into 5 principles/postulates: 1– Remember that the application is for the reviewer, not you, the applicant. 2– Communicate in stories just as the ancient Greek taught. 3– Make your Specific Aims story cohesive and leave no puzzling gaps. 4– Motivate the reviewer to keep reading by making your story resonate. 5– Accept the serendipity and noise that are inherent in the peer-review system.

We also closed with 4 key take-aways: Proposition 1– Make peace with imperfection and apply. Proposition 2– Contact your NIH SRO and PO for help. Proposition 3– Don’t make your reviewer/reader think. Proposition 4– Never forget the African proverb, “If the lion doesn’t tell its story, the hunter will”.

Our contribution has fortunately been very well received. Altmetric reports that our paper has reached the top 1% of the >25 million papers it tracks. Key to this reception was the kind enthusiastic support from scientists and clinicians like Drs. Marcelo Bonini [2], Sara Thomasy [3], Saad Bhamla [4], Degui Zhi [5], Fabien Maldonado [6], Marc Sommer [7] and Michael Chiang [8], and especially Drs. Padhu Pattabiraman [9] and Naftali Kaminski [10] as well as other generous commentators.

So, for the next 45 minutes (the time it takes to read our article) you may want to suspend what you think you know about peer review whether as an applicant or a reviewer and read our paper. Share it if you think it could be of help to other potential applicants through your favorite social media outlet. And of course, feedback is most welcome.

One final proposition which Epictetus would surely affirm: Be kind to yourself – it’s the fulcrum on which it all balances.
